# Oligodendrocytes as Regulators of Neuronal Networks during Early Postnatal Development

**DOI:** 10.1371/journal.pone.0019849

**Published:** 2011-05-12

**Authors:** Sandrine Doretto, Monica Malerba, Maria Ramos, Taruna Ikrar, Chisato Kinoshita, Claudia De Mei, Emanuele Tirotta, Xiangmin Xu, Emiliana Borrelli

**Affiliations:** 1 Department of Microbiology and Molecular Genetics and U904 INSERM/UCI, University of California Irvine, Irvine, California, United States of America; 2 Department of Anatomy and Neurobiology, University of California Irvine, Irvine, California, United States of America; National Institutes of Health, United States of America

## Abstract

Oligodendrocytes are the glial cells responsible for myelin formation. Myelination occurs during the first postnatal weeks and, in rodents, is completed during the third week after birth. Myelin ensures the fast conduction of the nerve impulse; in the adult, myelin proteins have an inhibitory role on axon growth and regeneration after injury. During brain development, oligodendrocytes precursors originating in multiple locations along the antero-posterior axis actively proliferate and migrate to colonize the whole brain. Whether the initial interactions between oligodendrocytes and neurons might play a functional role before the onset of myelination is still not completely elucidated. In this article, we addressed this question by transgenically targeted ablation of proliferating oligodendrocytes during cerebellum development. Interestingly, we show that depletion of oligodendrocytes at postnatal day 1 (P1) profoundly affects the establishment of cerebellar circuitries. We observed an impressive deregulation in the expression of molecules involved in axon growth, guidance and synaptic plasticity. These effects were accompanied by an outstanding increase of neurofilament staining observed 4 hours after the beginning of the ablation protocol, likely dependent from sprouting of cerebellar fibers. Oligodendrocyte ablation modifies localization and function of ionotropic glutamate receptors in Purkinje neurons. These results show a novel oligodendrocyte function expressed during early postnatal brain development, where these cells participate in the formation of cerebellar circuitries, and influence its development.

## Introduction

The function of neurons in the mammalian central nervous system (CNS) strongly relies on the presence of glial cells. Oligodendrocytes, in particular, have the role of generating myelin sheaths around most axons of the vertebrate CNS allowing a faster conduction of the nerve impulse. The role of oligodendrocytes in axonal support and myelin formation in the CNS is well documented [1], [2]. Myelination is nevertheless a specialized function of mature oligodendrocytes, leaving the role of these cells, during development, still only partially explored. More recently, novel functions have been attributed to NG2-positive oligodendrocyte precursors in the formation and stabilization of climbing fibers-Purkinje cell synapses [3] and in the maintenance of the Ranvier's node [1]. In addition, oligodendrocytes participate in the maintenance of axonal health and stability in the adult [4]. Myelin specific proteins have been shown to inhibit sprouting and regeneration of axons in lesioned nerve tissue [5], [6]. This inhibitory function of oligodendrocytes raises the question of whether it is specific of adult tissue. Alternatively, it may be a more general function initially required during development to direct and stabilize neuronal connections before myelination is initiated and it is then further refined in the adult to prevent aberrant connections.

We have previously shown that oligodendrocyte ablation severely affects cerebellar development. Evidence was obtained using the MBP-TK transgenic mouse line; in these mice, oligodendrocyte's death can be temporally induced by the systemic administration of the nucleoside analogue FIAU coupled to the transgenic expression of the viral thymidine kinase I gene (TK) in oligodendrocytes [7], [8], [9]. Using this system we showed that oligodendrocyte ablation during the first postnatal week results into a severe structural impairment of the cerebellar cortex. In particular oligodendrocyte-deprived cerebella were smaller and characterized by misaligned Purkinje cells with stunted dendritic trees and a reduced number of granule cells [10], [11]. Importantly, the recovery of oligodendrocytes and myelin, which follows the arrest of the ablation protocol, does not result into a complete reorganization of the cerebellar cortex [10]. These results strongly suggest that oligodendrocytes are an early and absolute requirement for the normal development of this structure. However, previous results were obtained after chronic FIAU treatments, which did not allow identifying the immediate consequences of oligodendrocyte ablation that lead to the defective development of the cerebellar cortex.

In this article, we addressed this point by analyzing cerebella from WT and MBP-TK mice during the first postnatal day immediately following oligodendrocyte ablation. Strikingly, we observed that ablation of oligodendrocytes, at postnatal day 1 (P1) thus before myelination is initiated, results into a very rapid increase of neurofilament staining in the white matter tract of MBP-TK mice. This increase appears dependent from a sprouting event, which is accompanied by alterations in the expression of molecules involved in axonal growth and guidance in Purkinje and granule cell neurons together with deregulation of ionotropic glutamate receptors. Altogether these results indicate that oligodendrocytes play a central role in the regulation of cerebellar circuits' formation. In addition, these results suggest that these cells have an important role during brain development, before the myelination process is started. This early oligodendrocyte regulatory function appears to be required to help restricting and directing axonal growth and synapse formation.

## Results

**Figure 1:**
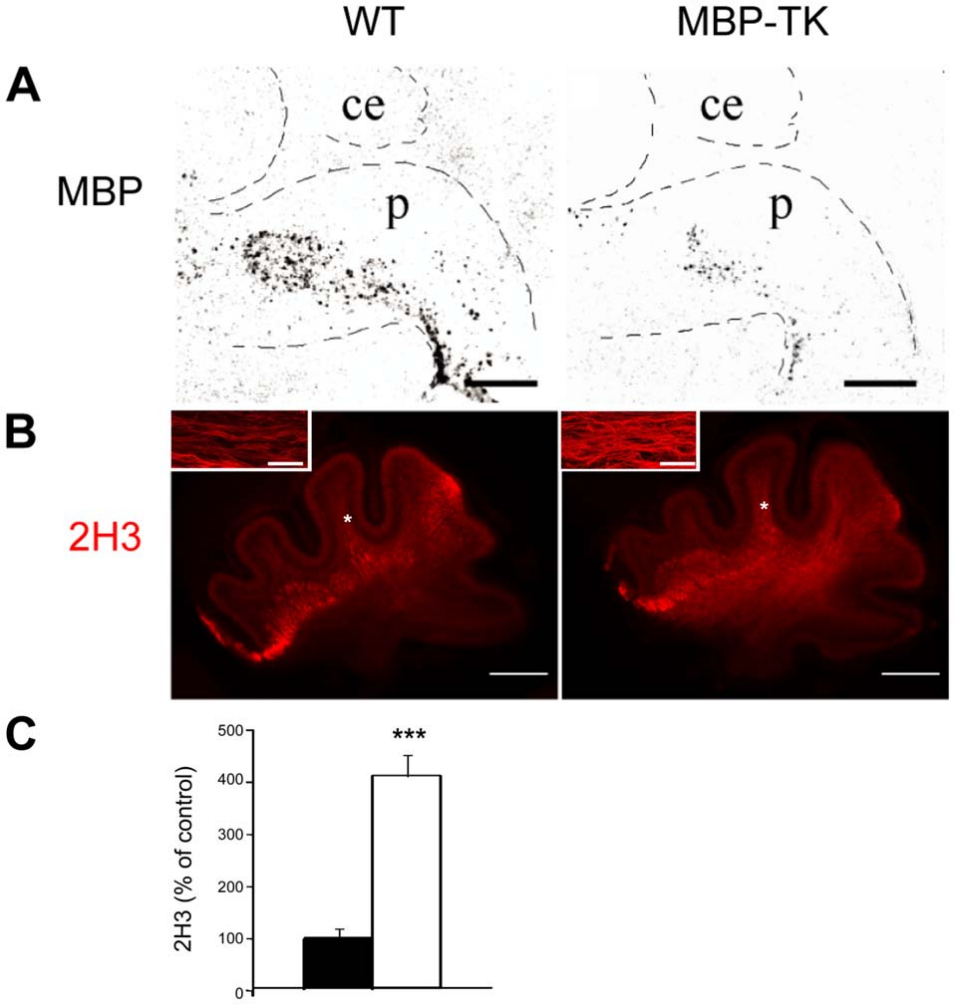
Sprouting of nerve fibers in MBP-TK mice after oligodendrocyte ablation. (A) MBP expression analyzed by in situ hybridization using a MBP specific riboprobe in WT (left panel) and MBP-TK (right panel) brain sections. Ce = cerebellum; p = pons. Scale bar: 500 µm. (B) Immunofluorescence analysis using an anti-neurofilament (2H3) antibody showing sprouting of nerve fibers in the cerebellar white matter tract of MBP-TK FIAU treated (right panel) compared to WT (left panel) brain sections. Scale bar: 200 µm. Insets show a magnification of the area marked by the asterisk in folia V (Scale bar: 10 µm). (C) Quantification of 2H3 fluorescence intensity in MBP-TK versus WT cerebella expressed as percentage of control. WT: black bar; MBP-TK: white bar. Values are mean ± SEM. Student's t-test: ***P<0.001.

In rodents, the process of myelination occurs essentially after birth and it is completed by the third postnatal week. Myelination in the CNS proceeds along a caudo-rostral axis; at postnatal day 1 (P1), mature oligodendrocytes expressing markers such as myelin basic protein (MBP) are visible in the spinal cord, while at the mRNA level, MBP^+^ cell bodies are found in the ventral region of the brain stem [12] (Figure 1A). This is the location from where afferent fibers to the cerebellum such as climbing fibers originate. Our previous studies had shown that removal of oligodendrocytes during the first postnatal weeks strongly perturb cerebellum development [11], [13]. However, the initial mechanisms generating the observed perturbation were not identified.

Here we analyze the physiological consequence of an acute ablation of oligodendrocytes at P1 on the developing cerebellum. To achieve oligodendrocyte ablation in vivo we used MBP-TK transgenic mice [9], [10], [11], [13]. The transgenic expression of herpes virus I thymidine kinase (TK) allows the inducible ablation of cell types in vivo, upon administration of the nucleoside analog FIAU [7], [8]. In the MBP-TK mouse model, TK is under control of the MBP promoter, thereby FIAU administration will induce death of dividing MBP^+^ oligodendrocytes. Experiments were performed using MBP-TK and wild type (WT) littermates treated with a single injection of FIAU (40 mg/kg, s.c.) at P1. To evaluate the primary effects of oligodendrocyte ablation, a time course of FIAU effects was performed. These experiments showed a maximal oligodendrocyte ablation 4 hours after the treatment; thus all experiments were performed at this time point. In situ hybridization using a MBP specific probe was utilized to assess and quantify oligodendrocyte death. Quantification of oligodendrocyte ablation by FIAU at P1, resulted into a maximal and severe 70% reduction (n = 6, *P*<0.0001) of MBP^+^ oligodendrocytes in MBP-TK mice, but not in WT littermates (Figure 1A). The remaining ∼30% is very likely composed by postmitotic oligodendrocytes, which are not any longer sensitive to FIAU.

Importantly, with the exception of oligodendrocytes no other cerebellar cell types appeared affected by the treatment in MBP-TK mice, as verified by the use of cell type specific markers (Figure S1).

Since in the adult, oligodendrocytes death results into sprouting of nerve fibers, we evaluated the effect of oligodendrocyte ablation on this parameter by analyzing sections from treated mice of WT and MBP-TK genotypes, using the antibody 2H3 directed against the (160 KDa) neurofilament protein. This experiment revealed ∼4 fold (n = 5, *P*<0.0001) increase of neurofilament staining intensity in MBP-TK versus WT treated cerebella (Figure 1B), particularly evident in the cerebellar white matter tract. Higher magnifications of images in the cerebellar white matter tract (inset in Figure 1B) showed the presence of higher number of intensely stained fibers in MBP-TK as compared to control mice. These results suggest that oligodendrocyte ablation in newborn mice, as in adult brain injuries, is associated with sprouting of nerve fibers.

The development of specific neural connections is a multifactorial process in which the interplay between different signals, acting at sequential developmental stages, provides the cues necessary for the guidance and growth of axonal projections. Semaphorin 3A (Sema3A) and Netrin-1 are secreted proteins that play a critical role in the guidance of developing axons in the cerebellum as in other brain regions [14], [15]. Sema3A is produced by Purkinje cells and has been shown to repel fibers [15], [16], while Netrin-1 is expressed in the external granular layer and attracts or repels fibers depending on the type of receptor expressed by the target [16]. Netrin-1 and its receptors [17] are critical elements during cerebellum development in the establishment of cerebellar boundaries, granule neurons migration and survival [18], [19], [20]. Both Netrin-1 and Sema3A are also regulators of axonal branching [16].

**Figure 2:**
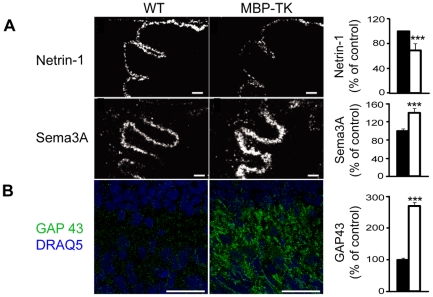
Oligodendrocyte ablation modifies the expression of axonal guidance molecules and neuronal plasticity markers. (A) In situ hybridization with Netrin-1 and Sema3A specific antisense riboprobes in MBP-TK and WT treated littermates in P1 cerebella. Scale bar 100 µm. Quantification (expressed as % of control) of Netrin-1 shows a decrease of its expression in granule cell precursors of the external granular layer; while a concomitant increase of Sema3A expressed by Purkinje neurons is observed in MBP-TK mice versus WT siblings. Values are mean ± SEM and analyzed by Student's t-test: ***P<0.001 (B) Double immunostainings using anti-GAP43 antibody (green) and DRAQ-5 (blue), Scale bar: 25 µm. MBP-TK animals have a higher GAP43 level compare to their WT littermates. Values are mean ± SEM and analyzed by Mann-Whitney U-test: ***P<0.001. WT: black bar; MBP-TK: white bar.

The observed increase of neurofilament-positive fibers in the white matter tract of MBP-TK mice, following oligodendrocytes, ablation brought us to investigate whether the expression of Netrin-1 and Sema3A might be influenced in areas targeted by cerebellar afferences. Strikingly, 4 hours after oligodendrocyte ablation, the pattern of expression of Sema3A and Netrin-1 throughout the cerebella of MBP-TK treated mice was profoundly altered as compared to treated WT littermates. Indeed, in situ hybridization experiments performed using Netrin-1 and Sema3A specific probes on sagittal cerebellar sections showed 31% reduction (n = 10, *P*<0.0001) of Netrin-1 in granule cell precursors located in external granular layer (EGL) (Figure 2 top panel); conversely 40% increase (n = 19, *P*<0.0001) of Sema3A was observed in Purkinje neurons (Figure 2 center panel) in MBP-TK treated mice as compared to WT treated littermates. The level of expression of Sema3A and Netrin-1 in WT untreated was identical to that of WT treated mice as well as in MBP-TK untreated cerebella (data not shown), thus indicating that the observed differences between WT and MBP-TK mice are specifically due to absence of oligodendrocytes in MBP-TK treated animals.

Next we analyzed the expression of proteins involved in dynamic cellular processes, such as neurite outgrowth, synaptic plasticity and nerve regeneration such as Growth-Associated Protein 43 (GAP43) [21], [22]. Quantifications of labeling intensity showed an increase of GAP43 stainings in MBP-TK (270.7±11.1%; n = 4, *P*<0.0005) versus WT treated mice (n = 6) (Figure 2 bottom panel). Interestingly, the increase of this protein parallels that of neurofilament (160 KDa) (2H3 staining) (Figure 1B).

**Figure 3:**
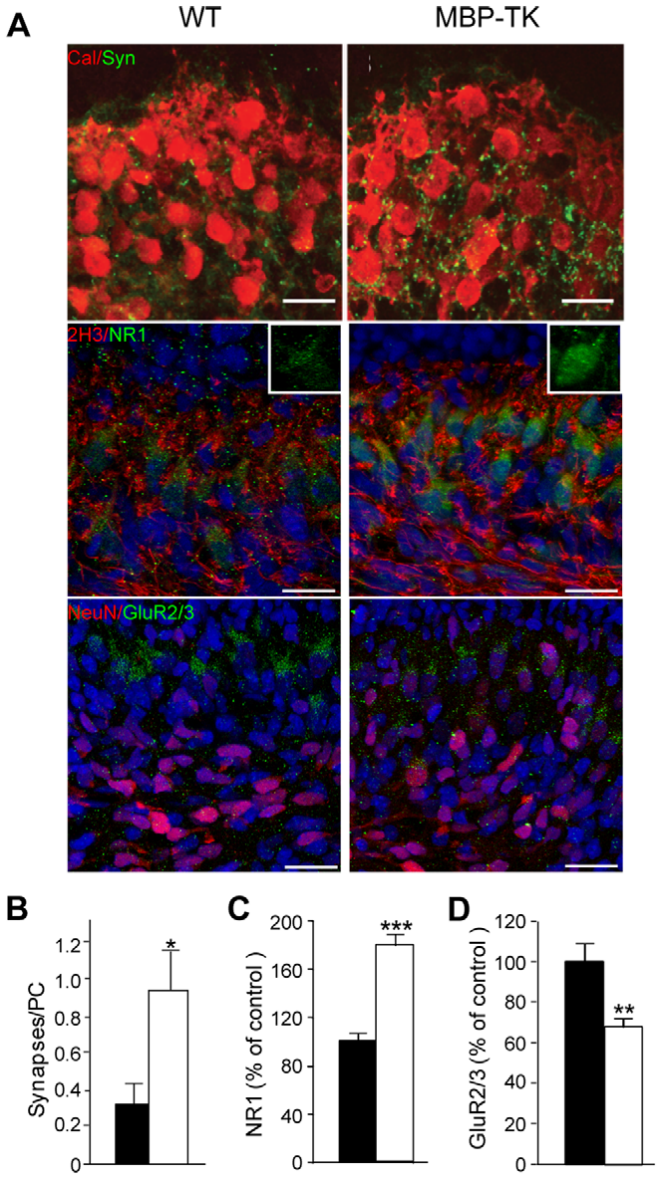
Oligodendrocyte ablation affects synapse formation and remodeling at P1. (A) Top panel: Double immunostaining with antibodies directed against calbindin (Cal, red) and synaptophysin (Syn, green) showed an important increase of synaptophysin staining in MBP-TK treated animals as compared to WT treated littermates. Middle panel: double immunostaining using antibodies directed against (160 KDa) neurofilament (2H3, red) and NMDAR subunit NR1 (green) showing differences in NR1 localization between WT and MBP-TK treated mice. Insets (scale bar 12.5 µm) show NR1 localization on PC soma and dendrites in the WT whereas in MBP-TK mice NR1 staining appears internalized. Bottom panel: double immunostaining directed against Neu N (red) and AMPAR subunit GluR2/3 (green) showing a decrease of GluR2/3 staining in MBP-TK treated animals. (B) Quantification of synapses number on PC's bodies performed on EM specimen at P1 (4 h after treatment) revealed a 3.2 fold increase of synapse number in MBP-TK versus WT PCs (n = 20 images, P<0.05). (C and D) Quantification of NR1 and GluR2/3 staining intensities in MBP-TK mice versus WT siblings, respectively. Black bar: WT, White bar: MBP-TK. Values are mean ± SEM. *P<0.05, **P<0.01, ***P<0.001. Scale bar 20 µm.

Immunostaining of cerebellar sections at P1, using antibodies directed against calbindin and synaptophysin revealed the presence of only few synaptophysin positive puncta around Purkinje cells soma in WT cerebella (Figure 3A). In contrast, analyses of MBP-TK treated cerebella sections revealed numerous synaptophysin positive puncta mostly located around Purkinje cells soma (Figure 3A) (225.5±25.6%; n = 6; *P*<0.005). These results suggest that as consequence of oligodendrocyte ablation, sprouting of afferent fibers might increase the number of synapses with cerebellar neurons in MBP-TK treated mice. Quantifications of synapse number per Purkinje cells were performed on electron microscopy (EM) images of WT and MBP-TK treated cerebella specimen (Figure 3B). 0.2±0.12 synapses were counted on WT Purkinje cell bodies at P1 whereas this number reached 0.95±0.2 in transgenic cerebella (n = 20, *P*<0.01). The increased synapse number found in MBP-TK cerebella can be correlated with the higher Sema3A and GAP43 expression, and their role in synaptic plasticity [23], [24], [25].

Since in P1 MBP-TK treated cerebella we observed modifications of arborization, synapse number and expression of presynaptic components, we aimed at investigating whether alterations of cerebellar afferent fibers could affect the physiology of Purkinje cells. Purkinje cells receive inputs from climbing fibers, which are glutamatergic [26]. Expression of several ionotropic glutamate receptors have been described in the cerebellar cortex [27], [28] and shown to play important roles during development [29]. In addition, N-methyl-D-aspartate receptors (NMDARs) have been reported to regulate synapse size and density during brain development [30]. In particular, NR1 expression has been visualized in Purkinje cells by different means [31], [32].

Immunostaining were thus performed on brain sections from WT and MBP-TK treated mice using antibodies directed against NR1, the obligatory subunit of NMDARs (Figure 3A). Interestingly, differences in the expression and localization of NR1 in Purkinje cells were observed between WT and transgenic P1 treated mice. In WT cerebella, NR1 staining showed the presence of isolated puncta located toward the external granular layer (EGL), and puncta accumulation on the Purkinje cell's soma (see inset Figure 3A). Surprisingly, NR1 staining in MBP-TK treated cerebella revealed a more diffused and stronger intracellular staining of Purkinje cells soma with several puncta (see inset Figure 3A), while we noticed absence of staining toward the EGL. Quantification of NR1 labeling intensity (Figure 3C) indicated a significant increase of NR1 staining in Purkinje cells from MBP-TK mice (178±9.5%; n = 6; *P*<0.001) as compared to those of WT treated littermates (n = 6). Similar induction was observed using an antibody directed against NR2A/2B subunits of the NMDA receptor (data not shown). During development, the ratio AMPA (α-amino-3-hydroxy-5-methyl-4-isoxazole propionic acid) to NMDA receptors mediated currents increases at excitatory synapses [29], [33]. We thus analyzed AMPA receptors expression in the cerebellum of WT and MBP-TK treated animals at P1 using an antibody directed against GluR2/3 receptors. At P1 GluR2/3 positive staining was principally observed at the level of Purkinje cells (68±4.3%; n = 6; *P* = 0.004) (Fig. 3A). Interestingly, GluR2/3 specific staining was reduced in the Purkinje cells of MBP-TK treated mice. These data indicate that axonal sprouting, induced by oligodendrocyte ablation at P1, leads to changes in the ratio of ionotropic NMDARs.

The observations reported above, led us to investigate whether the electrophysiological properties of cerebellar neurons and in particular Purkinje cells were affected. For this, we performed whole cell recordings from individual neurons from WT and MBP-TK treated mice. These analyses indicated no significant differences in the intrinsic properties of the age-matched cells between WT and MBP-TK cells (6 MBPTK cells, 15 WT cells) (Supplemental Figure S2). All the recorded cells were identified as Purkinje cells, for their localization in the Purkinje cell layer and for the presence of long axons projecting to deep cerebellar nuclei (Supplemental Figure S2B, inset). All the neonatal neurons recorded exhibited immature spiking patterns in response to strong depolarizing current injections. Recordings were performed at P1 4 hours after FIAU treatment. In addition, groups of animals injected at P1 were also analyzed in the following days until P5. The average resting membrane potentials for P1–P2 cells [36.7±0.8 mV (n = 13)] were more depolarized than P3–P5 cells [−51.6±1.9 mV (n = 6) (P<0.005)].

**Figure 4:**
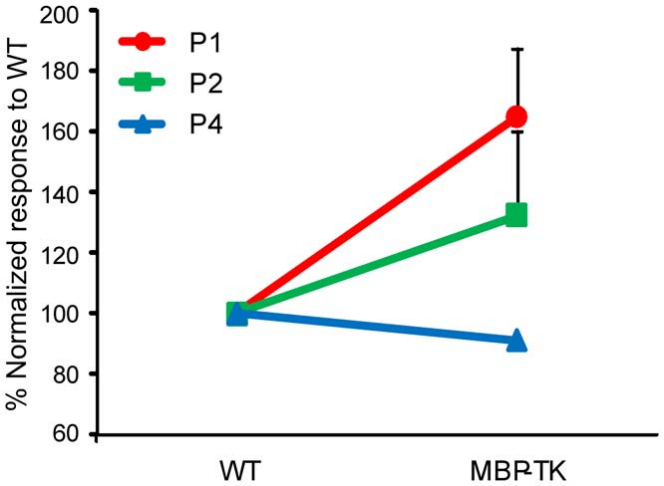
Normalized responses of MBP-TK versus WT slices. The MBP-TK data were acquired from the same batch of experiments as for WT littermates. Data are represented as mean ± SE. MBP-TK cerebellar neurons at P1–P2 show a trend toward increased excitability with respect to WT neurons; by P4 this trend is inversed and neurons are less excitable. The VSD response of P1 and P2 MBPTK slices was significantly higher than that of WT slices (P = 0.048, n = 6 slice pairs), while responses of P4 MBPTK slices were significantly lower than control slices (P = 0.03, n = 4 slice pairs). Note: the error bar for the P4 responses is small and thereby not visible in the graph.

The difference observed in NMDA receptor expression/localization led us to analyze the responses of neonatal cerebellar neurons to glutamate uncaging, via laser scanning photostimulation [34], [35]. Both whole-cell (3 cells) and local field potential recordings (5 slices) in neonatal cerebellar slices revealed excitatory neuronal responses to glutamate uncaging. As shown in Supplemental Figure S3, even in the early developmental stages, cerebellar cortical neurons had established local circuit connections as indicated by clear EPSCs to the recorded neurons from the photostimulated locations. Next, we characterized the neuronal population responses using a newly developed functional mapping technique, which measures neuronal excitability by detecting photostimulation-evoked population responses through voltage sensitive dye (VSD) imaging. [35] (Supplemental Figure S4). Our imaging data indicated that MBPTK slices from FIAU treated mice exhibited higher excitability as reflected by stronger evoked VSD responses at P1 and P2, compared to WT slices (P = 0.048, N = 6 slice pairs) (Figure 4). Specifically, on average, the normalized MBPTK slice responses to WT control slices were 164.9±22.6% (mean ± SE), and 132±34%, for P1 and P2 (n = 3 slice each), respectively (Figure 4).

**Figure 5:**
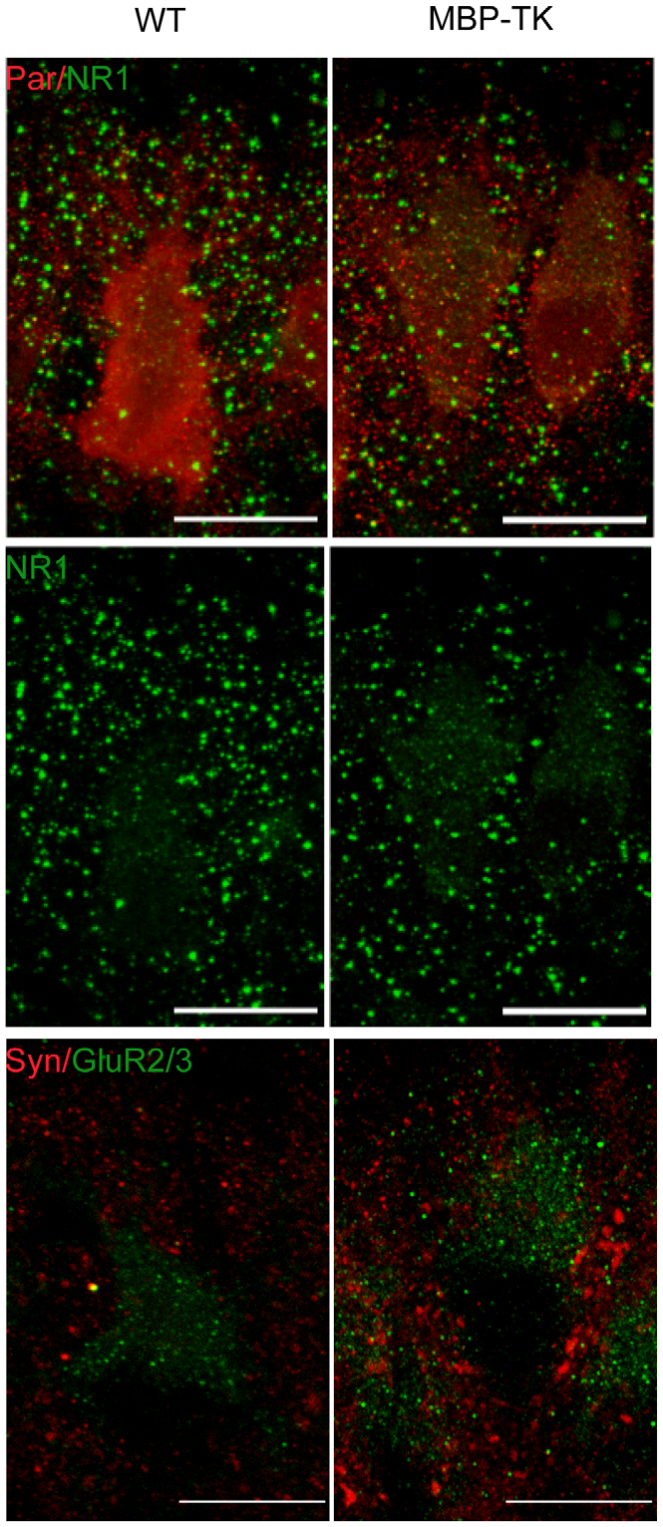
Oligodendrocyte ablation at P1 leads to modifications of glutamate receptors levels in Purkinje neurons. Double immunostaining were performed in animals treated only at P1 and analyzed at P5 with antibodies directed against parvalbumin (Par, red), NMDAR subunit NR1 (green), GluR2/3 and synaptophysin (syn). Scale bar: 20 µm.

**Figure 6:**
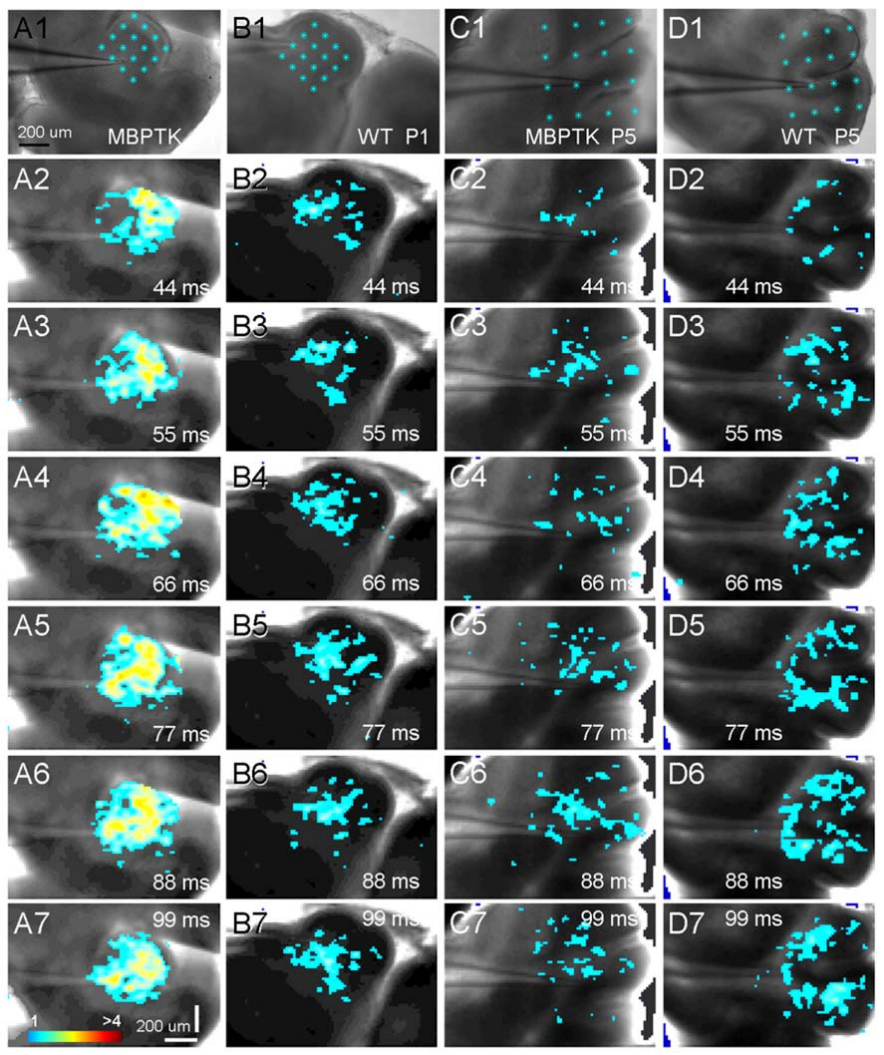
Neuronal population responses in WT and MBP-TK treated cerebellar cortical slices. (A, B) VSD image frames in response to multiple site photostimulation (laser duration: 2 ms; power: 20–24 mW) in WT and MBP-TK treated cerebellar cortical slices (P1), respectively. These image frames are pooled from the image data of all photostimulation sites, as indicated in the reference images. Time progresses from top to bottom in the column, and color code is used to indicate VSD signal amplitudes expressed as standard deviations (SD) above the mean baseline. Similarly, C and D are the VSD image frames in response to multiple site photostimulation (laser duration: 2 ms; power: 20–24 mW) in the wild type and MBP-TK treated cerebellar cortical slices (P5), respectively. MBP-TK treated slices exhibited overall higher excitability in P1–P2 than P4–P5, as reflected by stronger evoked VSD responses. WT slices tended to have a more similar excitability across P1–P5.

Previous studies had shown that remyelination takes place in MBP-TK treated mice [10]. Taking advantage of the inducible toxic nature of the TK system, we analyzed whether the alterations on nerve fibers and glutamate receptors expression caused by oligodendrocyte ablation at P1 would be reversible. Thus, groups of WT and MBP-TK littermates treated with FIAU at P1 were allowed a few days for recovery and analyzed. Quantification of synaptophysin staining intensity showed that the level of expression of this protein remained elevated with respect to WT littermates and similar to P1 values (249.4±16%; n = 5; p<0.001). In sharp contrast, at P5 the expression of NR1 (Figure 5) in MBP-TK was reduced with respect to WT treated mice (84±0.8%); this difference is opposite to that observed at P1 (Figure 3) where the MBP-TK mice showed an increased staining intensity for NR1 as compared to WT littermates (178±9.5%). Conversely, GluR2/3 labeling intensity at P5 (133.24±17.8%) was higher with respect to the staining levels observed at P1 (68±4.3%) with respect to WT siblings (Figure 5). Imaging experiments showed that the excitability of MBP-TK slices from animals treated at P1 and analyzed at P4 was reduced as compared to WT slices from littermates similarly treated (Figures 4, 6).

## Discussion

The role of myelin as regulator of axonal growth is well characterized in the adult CNS [5]. Oligodendrocytes are the glial myelin-producing cells of the CNS. The role of these cells during CNS development is less known; oligodendrocyte precursors appear during embryonal development [36], but myelination starts only after birth. Previous studies have shown that ablation of oligodendrocyte during the first postnatal weeks leads to major impairments in cell number, structure and function of the cerebellar cortex [9], [11], [13]. The cerebellum develops essentially during the postnatal period and thereby represents an interesting model system to analyze the potential involvement of oligodendrocyte in development.

In this article, we aimed at characterizing the early events that follow oligodendrocytes ablation in order to determine the impact of these cells on cerebellum postnatal development. Interestingly, loss of oligodendrocytes at P1, promotes an immediate reaction in cerebellar nerve fibers, detected by a stronger immunoreactivity to neurofilament antibodies (Figure 1). Analyses of cerebellar sections, at this time point show the presence of multiple fibers in the cerebellum of MBP-TK mice with respect to WT, suggesting sprouting of nerve fibers. This effect has been observed in adult brains following injury; however, in the adult the axonal growth is very limited due to the presence of myelin inhibitory factors and lack of growth supporting molecules [37], [38]. At P1, the presence of oligodendrocytes is restricted to the brain stem [12] and these cells are still immature and do not produce yet proteins, which normally inhibit neurite growth [37]. Thus, it is interesting to note that oligodendrocytes can regulate axonal growth at distance, suggesting that either physical contacts between oligodendrocytes and neurons or diffusible factors from these or other glial cells [5], [39], [40] might affect neurite growth at distal sites. Our results also suggest that sprouting of cerebellar fibers is independent from myelin destruction. As consequence of OL ablation, we also found outstanding changes in the expression of pre- and post-synaptic proteins as well as of neuronal plasticity markers in the cerebellum.

Absence of MBP+ oligodendrocyte at P1 impairs the establishment of proper synaptic contacts at distal sites, which is likely to be resulting from sprouting of cerebellar fibers. In particular, we observed an outstanding increase of GAP 43 [22], a molecule strongly involved in neurite outgrowth and plasticity [24] in afferents to cerebellar neurons. As consequence, the physiology of Purkinje cells and granule neuron precursors is affected as determined by alteration of the expression of proteins required for the guidance and maturation of neuronal circuitries. The rapid alterations in the expression of these proteins identify them as immediate responsive genes, further supporting their involvement in the formation of neuronal circuitries [14], [15], [16], [21]. Moreover, the increase of synaptic markers observed in MBP-TK mice cerebella is correlated with the increase in Sema3A expression, which is in agreement with findings that these molecules have synaptogenic properties [23].

During early postnatal development axons of cerebellar afferents, the climbing and mossy fibers, start to make excitatory synaptic connexions with Purkinje and granule neurons, respectively [41], [42]. In particular, in newborn mice Purkinje receive multiple innervations by the climbing fibers [41], [42], which as development proceeds are progressively eliminated to attain a mono-innervation by the end of the third postnatal week [43]. Glutamate signaling also regulates the process of synapse elimination. Indeed, NMDAR blockade prevent the regression of supernumerary climbing fiber synapses in Purkinje cells [44].

In our experiments, we found that in the Purkinje cells of P1 MBP-TK treated cerebella the localization of the obligatory NMDAR subunit NR1 is changed and its expression increased; conversely the expression of AMPA receptors in diminished. These results are in agreement with reports showing changes in the NMDA/AMPA ratio during development [29].

The increased expression of axonal growth and synaptic plasticity markers observed at P1 in MBP-TK slices leads to an increased glutamate excitability with respect to the WT slices. Interestingly, this higher excitability is not maintained with time; indeed, when animals treated at P1 are analyzed after four days of recovery from oligodendrocyte ablation, we observed a reduction of NR1 staining intensity and a corresponding reduced response to glutamate activation. Considering that VSD imaging of evoked activation reflects the combined contributions of direct neuronal responses and postsynaptic mediated signals, oligodendrocyte ablation at P1 severely affects maturation of cerebellar circuitries in an irreversible manner. Our studies suggest that oligodendrocytes play an important role on axonal growth and participate in the formation of neuronal circuitries in the cerebellum. Absence of this control results into altered glutamate receptors expression in Purkinje cells, which affects the normal development of the cerebellar cortex [2], [10], [11], [13]. We have previously reported that chronic FIAU treatment of MBP-TK mice during the first three postnatal days results into a reduction of the cerebellum size, due to a strong reduction of mature granule neurons in the internal granular layer [11], [13]. It was proposed that the decrease of Sonic Hedgehog (Shh) [13] produced from Purkinje neurons after oligodendrocyte ablation, affects granule neurons' proliferation. Here, we show that just few hours after oligodendrocyte ablation, the expression of Netrin-1 is strongly reduced in granule neurons, which might additionally impair their maturation and migration from the external to the internal granular layer [18].

It is tempting to propose a mechanism by which oligodendrocyte ablation induces important changes in neurite outgrowth and plasticity of cerebellar afferent fibers, as manifested by the increased expression of GAP43 and synaptic markers. Increased synaptic contacts on Purkinje neurons induces an upregulation of chemorepellent molecules, such as Sema 3A and at the same time down-regulation of proteins required for granule neurons proliferation and maturation such as Shh [13]. As consequence, granule neurons physiology is also affected as shown by Netrin-1 down-regulation at P1, which impairs maturation and exit of these neurons from the external granular layer leading to their death in the following days [13]. As result, only few mature granule neurons are present in the internal granular layer, leading to reduced interactions between granule neuron and Purkinje cells [11], [13].

Overall, these findings reveal an unappreciated role of oligodendrocytes precursors during the earlier postnatal brain development. Our study shows that removal of oligodendrocytes strongly affects axonal targeting and refinement of nerve connections and that these effects are observed even before myelination is completed. It would be of interest to assess, in future studies, whether ablation of oligodendrocyte precursors performed during the late phase of embryogenesis might further affect structural plasticity. It thus appears that the adult oligodendrocyte function as inhibitor of axonal sprouting and regeneration is not only a feature of the adult brain, but it is a more general function also required during the early postnatal stage of brain development during formation of neuronal circuitries.

## Materials and Methods

All animal work described in the study was carried out in accordance with the guidelines of the National Institutes of Health regarding the care and use of animals for experimental procedures, and approved by the Institutional Animal Care and Use Committee (IACUC) of the University of California Irvine (protocol #2006-2667).

### Transgenic mice

MBP-TK mice were generated in the laboratory and previously described [9]. WT and MBP-TK siblings are in a C57/Bl6 background. WT controls always belong to the same litter than transgenic mice and received the same treatment.

### Treatments

FIAU [1-(2-deoxy-2-fluoro-ß–arabinofuranosyl)-5-iodouracil] (generously provided by Bristol-Myers Squibb, Wallingford, CT) (40 mg/kg of body weight) were dissolved in saline solution and injected subcutaneously at P1 (the day of birth is referred to as postnatal day P0). Animals were killed 4 hrs after the injection or at P5; for electrophysiological recordings, animals were injected at P1 and then analyzed 4 hours later, or in following days until P5.

### Immunofluorescence and Quantifications

After deep anesthesia, mice were perfused transcardially with 4% paraformaldehyde in phosphate buffer (pH 7.4, 0.1 M). Brains were postfixed, and 100-µm vibratome sections were made. Sections were blocked in 5% normal goat serum in PBS, 0.3% Triton X-100. The antibodies used were: mouse anti-neurofilament (160 KDa) 2H3 (Developmental Studies Hybridoma Bank, The University of Iowa, Iowa City, USA) (1∶10), mouse anti-synaptophysin (Sigma) (1∶1000); mouse anti-parvalbumin (Chemicon) (1∶1000), rabbit anti-calbindin D-28k (Swant, Bellinzona, Switzerland) (1∶1,000), rabbit anti-GAP43 (Chemicon) (1∶500), rabbit anti-NR1 (Chemicon) (1∶200), mouse anti-NeuN (Chemicon) (1∶200), rabbit anti-PAX2 (Zymed) (1∶400), rat anti-PDGF-alpha Receptor (Pharmingen) (1∶200), rabbit anti-NG2 (Chemicon) (1∶400), rabbit anti-GluR2/3 (Upstate Biotechnology) (1∶1000), mouse anti-BrdU (Sigma) (1∶1000), mouse anti-Nestin (Chemicon) (1∶200). Fluorescent secondary antibodies used were: goat anti-mouse and goat anti-rabbit IgG conjugated with Alexa Fluor 488 (1∶800) or Cy3 (1∶200) (Molecular Probes). Nuclei were stained with DRAQ-5[1,5-Bis[[2-(dimethylamino)ethyl]amino]-4,8-dihydroxyanthracene-9,10-dione] (Biostatus Limited, Leicestershire, UK). Immunolabelled sections were examined with a Leica confocal microscope SP5 (DMRE, Leica, Heidelberg, Germany). Controls were always performed by omitting primary antibodies. At least three different animals per genotype and time point were analyzed, and experiments were repeated three times. Tissue samples were stained with antibodies and analyzed by confocal microscopy with the same exposure/gain. Stacks of images (10 µm) for each experiment and genotype were compressed, background subtracted and a threshold was applied on each image so that ∼15% of the total signal was eliminated as background. Fluorescence intensity was quantified in the regions of interest of images taken at 100z2 magnification. Intensity of fluorescence is evaluated as pixels/µm^2^. The Leica SP5 software LAS AF was used for quantification.

TUNEL experiments were performed on brain cryosections post-fixed in 1% paraformaldehyde in PBS, using the In Situ Cell Death Detection Kit (Roche, Germany) and dUTP-coupled with Alexa Fluor 488 (Molecular Probes).

### Electron microscopy

After deep anesthesia, mice received an intracardiac perfusion of 3% glutaraldehyde and 2% paraformaldehyde in 0.1 M cacodylate buffer, pH 7.4. Optic nerves and brain stem regions were removed and kept overnight at 4°C in the same fixative. Tissues were post-fixed in 1% osmium tetroxide at 4°C for 2 hour in 0.1 M phosphate buffer, pH 7.4, followed by dehydration in graded ethanol baths, infiltrated with propylene oxyde and embedded in Epon. Semithin sections (1 µm) were stained with toluidine blue. Ultrathin sections (70 nm) were contrasted with 5% uranyl acetate and lead citrate and examined by electron microscopy (EM) (Morgagni 268, FEI) at 2800× magnification.

In situ hybridization was performed as previously described [9]. 35S-labeled RNA probes encoding MBP, Semaphorin-3A and Netrin-1 sense and antisense riboprobes were synthesized using T3, T7 or SP6 polymerase in the presence of cytidine 5′-[35S] thiotriphosphate (10 mCi/ml, Amersham), according to the supplier's directions (Stratagene, Biolabs). After probe hybridization, slides were coated with Kodak NTB2 emulsion and stored at 4°C. Emulsions were finally developed in Kodak 19 and tissues were counter-stained with Toluidine Blue. Silver precipitates representing the radiolabelling were visualized in dark and bright field images. The quantification of expression was performed on a fixed surface from different brain sections calculating the mean intensity of pixels in dark field gray-scale images.

#### Electrophysiology, photostimulation and voltage sensitive dye (VSD) imaging

Seventeen WT and 9 MBPTK FIAU treated mice (from P1 to P5) were used in the experiments. To prepare living brain slices, animals were deeply anesthetized with Nembutal (>100 mg/kg, i.p.), rapidly decapitated, and their brains removed. Parasaggital cerebellar cortical slices were cut 400 µm thick with a vibratome (VT1200S; Leica Systems, Germany) in sucrose-containing artificial cerebrospinal fluid (CSF) (in mM: 85 NaCl, 75 sucrose, 2.5 KCl, 25 glucose, 1.25 NaH2PO4, 4 MgCl2, 0.5 CaCl2, and 24 NaHCO3). Slices were first incubated in sucrose-containing ACSF for 30 minutes to 1 hour at 32°C, and after the initial incubation period, transferred to recording ACSF (in mM: 126 NaCl, 2.5 KCl, 26 NaHCO3, 2 CaCl2, 2 MgCl2, 1.25 NaH2PO4, and 10 glucose) for the dye staining at room temperature. Slices were stained for 1 hour in a staining chamber containing ACSF with 0.02 mg/ml of an oxonol dye, NK3630 (Nippon Kankoh-Shikiso Kenkyusho Co., Ltd., Japan), and then maintained in regular ACSF before use. Throughout the incubation, staining and recording, the slices were continuously bubbled with 95% O2–5% CO2.

Slices were visualized with an upright microscope (BW51X; Olympus, Tokyo, Japan) with infrared differential interference contrast optics. Electrophysiological recordings, photostimulation, and imaging of the slice preparations were done in a slice perfusion chamber mounted on a motorized stage of the microscope. At low magnification (4× objective lens, 0.16 NA; UPlanApo; Olympus), laminar and cytoarchitectonic features of brain slices were visualized under infrared bright-field transillumination; and the slice images were acquired by a high-resolution digital CCD camera (Retiga 2000, Q-imaging Inc, Austin, TX). Digitized images from the camera were used for guiding and registering photostimulation sites in cerebellar cortical slices.

To perform patch recording, cells were visualized at high magnification (60× objective, 0.9 NA; LUMPlanFl/IR; Olympus). Neurons were patched with borosilicate electrodes and recorded at room temperature in the whole-cell or loose-seal mode. The patch pipettes (4–6 MΩ resistance) were filled with an internal solution containing 126 mM K-gluconate, 4 mM KCl, 10 mM HEPES, 4 mM ATP-Mg, 0.3 mM GTP-Na, 10 mM phosphocreatine (pH 7.2, 300 mOsm). The electrodes also contained 0.5–1% biocytin for cell labeling and morphological identification. Resting membrane potentials were measured immediately after electrodes broke into the cells following formation of a gigaohm seal, and current pulses were injected to examine each cell's basic electrophysiological properties. Data were acquired with a Multiclamp 700B amplifier (Molecular Devices, Sunnyvale, CA), data acquisition boards (models PCI MIO 16E-4 and 6713; National Instruments, Austin, TX), and custom modified version of Ephus software (Ephus, available at https://openwiki.janelia.org/). Data were filtered at 2 kHz using a Bessel filter and digitized at 10 kHz and stored on a computer. Once stable whole-cell recordings were achieved with good access resistance (usually <20 MΩ), the microscope objective was switched from 60× to 4× for laser scanning photostimulation. The same low-power objective lens was used for delivering the UV flash stimuli.

Stock solution of MNI-caged-l-glutamate (4-methoxy-7-nitroindolinyl-caged l-glutamate, Tocris Bioscience, Ellisville, MO) was prepared by dissolving MNI-glutamate in distilled water and stored in 50 µl aliquots at −20°C for up to several weeks. An aliquot was added to 20–25 ml of circulating ACSF for a concentration of 0.4 mM caged glutamate.

Our laser scanning photostimulation and imaging system described in detail previously [35]. Briefly, a laser unit (model 3501; DPSS Lasers, Santa Clara, CA) was used to generate 355 nm UV laser for glutamate uncaging. The laser beam was 1.5 mm in diameter and directed through the optical path of our system. Short durations of laser flashes (e.g., 1–3 ms) were controlled by using an electro-optical modulator (i.e., pockels cell) (Conoptics, Danbury, CT) and a mechanical shutter (Uniblitz; Vincent Associates, Rochester, NY). Various laser stimulation positions could be achieved through galvanometers-driven XY scanning mirrors (Cambridge Technology, Cambridge, MA), as the mirrors and the back aperture of the objective were in conjugate planes, translating mirror positions into different scanning locations at the objective lens focal plane. A dual camera port was used to couple the Q-imaging camera and the laser scanning photostimulation system to a MiCAM02 fast imaging system (SciMedia USA Ltd, Costa Mesa, CA) for voltage sensitive dye imaging. Optical recording of VSD signals was performed by the MiCAM02 system with a sampling rate of 2.2 ms per frame (frame resolution 88 (w)×60 (h) pixels). Under the 4× objective, the imaging field covered the area of 1.28×1.07 mm2 with a spatial resolution of 14.6×17.9 µm/pixel. The trials were obtained every 8 seconds and the recording periods were 1000 frames for each photostimulation trial. VSD images were smoothed by convolving images with a Gaussian spatial filter (kernel size: 3×3 pixels; ∂size: 1×1 pixel) and a Gaussian temporal filter (kernel size: 3 frames; δ size: 1 frame). Signal amplitudes were expressed as standard deviations (SD) above the mean baseline signal for display and quantification. Images were displayed and initially analyzed using an acquisition and analysis software (BV-Analyzer; BrainVision, Tokyo, Japan). Further quantification and measurements were performed with custom-made Matlab Programs.

As for quantitative analysis of evoked activation in image frames, the mean and standard deviation of the baseline activity of each pixel across the 50 frames preceding photostimulation was first calculated, and then activated pixels were measured. The activated pixel was empirically defined as the pixel with the amplitude ≥1 SD above the mean of the corresponding pixel's amplitude preceding the stimulation (equivalent to the detectable signal level in the original VSD maps of ΔI/I %). The overall activation size in image frames was defined as the fraction of activated pixels, expressed as a percentage of the image frame size.

### Statistical Analysis

Results were analyzed by the Mann-Whitney *U* test, student t-test or ANOVA followed by the appropriate post hoc comparisons (*P*<0.05 was considered statistically significant).

## Supporting Information

Figure S1 **Oligodendrocyte ablation at P1 does not affect cerebellar cell types in MBP-TK treated mice.** MBP-TK mice treated with a single injection of FIAU at P1 do not show any cerebellar abnormality. The following parameters and cell markers were analyzed in MBP-TK and WT treated cerebella: (A–B) presence of apoptotic cells was analyzed by Tunel stainings; (C–D) presence of mitotic cells by BrdU staining; (E–F) Purkinje and granule neurons were visualized by Calbindin and NeuN antibodies, respectively; (G–H) interneurons were visualized using anti-Pax2 antibodies; (I–J) anti-Nestin antibodies were used to visualize astrocytes (Bergmann glia); (K–L) anti-PDGFαR and anti-NG2 antibodies were used to visualize OL precursors. These analyses did not reveal any difference between MBP-TK (B,D,F,H,J,L) and WT (A,C,E,G,I,K) cerebella, 4 hours after the first FIAU injection. Scale bar: 50 µm.
(TIF)

Figure S2 **Single cell recordings from the neonatal cerebellar cortex.** (A) Parasaggital slice of the cerebellum from a P1 WT mouse, with the small white square indicating the cell-recording site. (B) High-magnification image of the recording site with the recorded neuron identified as a PCs that had a long axon reaching deep into the cerebellum (see the inset). (C) Cell's responses to intra-somatic current injections. The cell's resting membrane potential was −34.7 mV. D–F similar formatted data than in A–C obtained from a P1 MBP-TK treated mouse. The cell's resting membrane potential was −30 mV.
(TIF)

Figure S3 **Examination of the responses of the neonatal cerebellar neurons to glutamate uncaging via laser scanning photostimulation.** (A) P2 WT cerebellar cortical slice image with the superimposed 5×5 photostimulation sites spaced at 75 µm apart. The insert shows the recorded neuron's intrinsic responses to intrasomatic current injections. (B) Data traces of the recorded neuron at the current clamp mode in response to laser photostimulation (1 ms, 24 mW) at the stimulus locations shown in A. A small red circle indicates the recorded cell body location. Note that the neuron had large potential depolarizations at sites 12 and 17. (C) Photostimulation-evoked response map from the locations as shown in B, while the cell was held at −40 mV at the voltage clamp mode to detect inward excitatory synaptic currents (EPSCs). While traces of 12 and 17 show predominantly direct response to glutamate uncaging, data traces of 18, 19, 23–25 (pointed by the arrowheads) illustrate clear EPSCs to the recorded neurons from the photostimulated locations.
(TIF)

Figure S4 **Example of voltage sensitive dye imaging of neuronal population responses evoked by laser photostimulation.** A1 is a reference image showing the cerebellar parasaggital slice from a P1 MBP-TK treated mouse, with the laser phostimulation site (indicated by the cyan dot). B1–B11 are sequences of VSD image frames in response to photostimulation (laser duration: 2 ms; power: 24 mW) in the lateral portions of the cerebellar cortical slice. The VSD images were acquired through the 4× objective at the rate of 2.2 ms/frame during the experiment, and are displayed once every 11 ms. Time progresses from left to right in the rows, and color code is used to indicate VSD signal amplitudes expressed as standard deviations (SD) above the mean baseline signal. The map pixels with amplitudes ≥1.1 SD are plotted and included for further quantification (see the Methods for details). Warmer colors indicate greater excitation. The site of photostimulation can be identified by the laser excitation artifact (the blue spot) in the initial frame of the sequences. Note that the CCD camera images have a slightly different aspect ratio. Under the 4× objective, the camera covers an area of 1.28 (w)×1.07 (h) mm2, with a spatial resolution of 14.6 (w)×17.9 (h) µm/pixel.
(TIF)
