# Supplementary figures and images for: Oligodendrocytes as Regulators of Neuronal Networks during Early Postnatal Development
Source: PLoS One. 2011 May 12;6(5):e19849. doi: 10.1371/journal.pone.0019849 (PMC3093406; doi:10.1371/journal.pone.0019849)

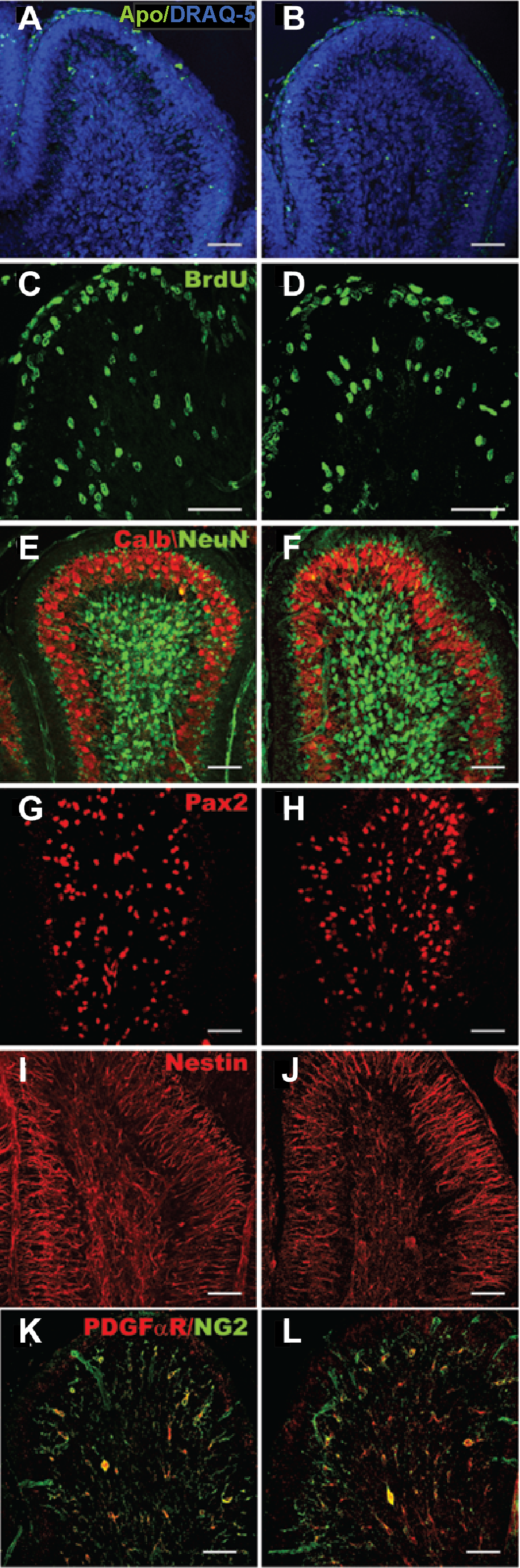

Supplement: Figure S1 — Oligodendrocyte ablation at P1 does not affect cerebellar cell types in MBP-TK treated mice. MBP-TK mice treated with a single injection of FIAU at P1 do not show any cerebellar abnormality. The following parameters and cell markers were analyzed in MBP-TK and WT treated cerebella: (A–B) presence of apoptotic cells was analyzed by Tunel stainings; (C–D) presence of mitotic cells by BrdU staining; (E–F) Purkinje and granule neurons were visualized by Calbindin and NeuN antibodies, respectively; (G–H) interneurons were visualized using anti-Pax2 antibodies; (I–J) anti-Nestin antibodies were used to visualize astrocytes (Bergmann glia); (K–L) anti-PDGFαR and anti-NG2 antibodies were used to visualize OL precursors. These analyses did not reveal any difference between MBP-TK (B,D,F,H,J,L) and WT (A,C,E,G,I,K) cerebella, 4 hours after the first FIAU injection. Scale bar: 50 µm. (TIF) [file pone.0019849.s001.tif]

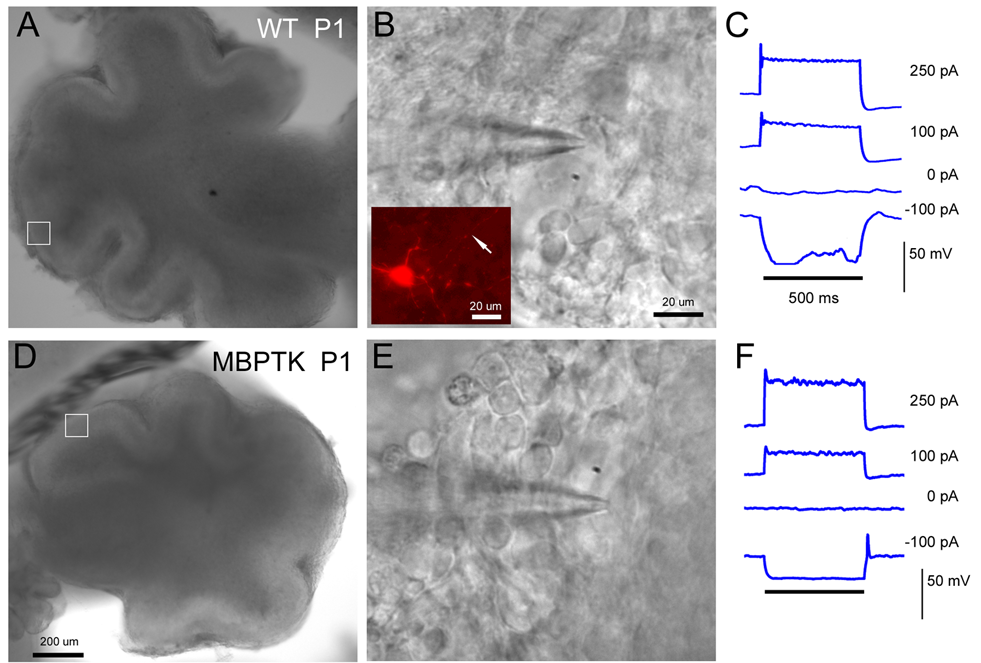

Supplement: Figure S2 — Single cell recordings from the neonatal cerebellar cortex. (A) Parasaggital slice of the cerebellum from a P1 WT mouse, with the small white square indicating the cell-recording site. (B) High-magnification image of the recording site with the recorded neuron identified as a PCs that had a long axon reaching deep into the cerebellum (see the inset). (C) Cell's responses to intra-somatic current injections. The cell's resting membrane potential was −34.7 mV. D–F similar formatted data than in A–C obtained from a P1 MBP-TK treated mouse. The cell's resting membrane potential was −30 mV. (TIF) [file pone.0019849.s002.tif]

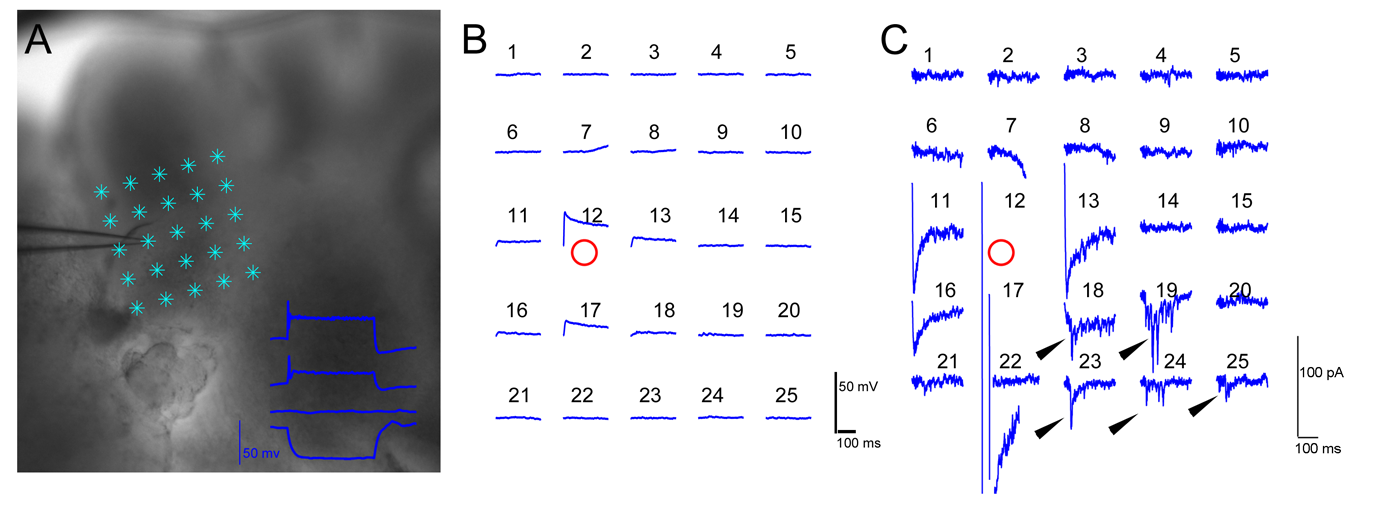

Supplement: Figure S3 — Examination of the responses of the neonatal cerebellar neurons to glutamate uncaging via laser scanning photostimulation. (A) P2 WT cerebellar cortical slice image with the superimposed 5×5 photostimulation sites spaced at 75 µm apart. The insert shows the recorded neuron's intrinsic responses to intrasomatic current injections. (B) Data traces of the recorded neuron at the current clamp mode in response to laser photostimulation (1 ms, 24 mW) at the stimulus locations shown in A. A small red circle indicates the recorded cell body location. Note that the neuron had large potential depolarizations at sites 12 and 17. (C) Photostimulation-evoked response map from the locations as shown in B, while the cell was held at −40 mV at the voltage clamp mode to detect inward excitatory synaptic currents (EPSCs). While traces of 12 and 17 show predominantly direct response to glutamate uncaging, data traces of 18, 19, 23–25 (pointed by the arrowheads) illustrate clear EPSCs to the recorded neurons from the photostimulated locations. (TIF) [file pone.0019849.s003.tif]

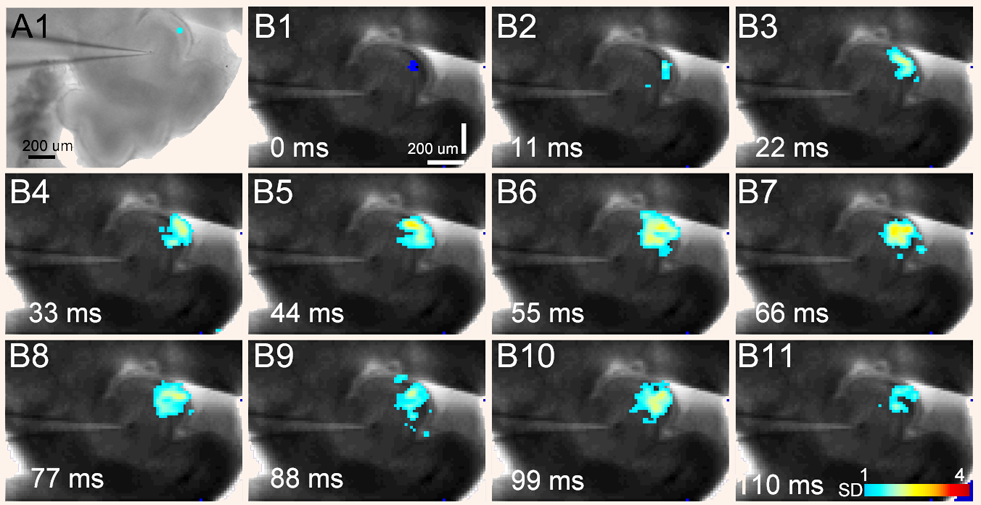

Supplement: Figure S4 — Example of voltage sensitive dye imaging of neuronal population responses evoked by laser photostimulation. A1 is a reference image showing the cerebellar parasaggital slice from a P1 MBP-TK treated mouse, with the laser phostimulation site (indicated by the cyan dot). B1–B11 are sequences of VSD image frames in response to photostimulation (laser duration: 2 ms; power: 24 mW) in the lateral portions of the cerebellar cortical slice. The VSD images were acquired through the 4× objective at the rate of 2.2 ms/frame during the experiment, and are displayed once every 11 ms. Time progresses from left to right in the rows, and color code is used to indicate VSD signal amplitudes expressed as standard deviations (SD) above the mean baseline signal. The map pixels with amplitudes ≥1.1 SD are plotted and included for further quantification (see the Methods for details). Warmer colors indicate greater excitation. The site of photostimulation can be identified by the laser excitation artifact (the blue spot) in the initial frame of the sequences. Note that the CCD camera images have a slightly different aspect ratio. Under the 4× objective, the camera covers an area of 1.28 (w)×1.07 (h) mm2, with a spatial resolution of 14.6 (w)×17.9 (h) µm/pixel. (TIF) [file pone.0019849.s004.tif]
